# Protective Effects of Picropodophyllotoxin on CLP-Induced Sepsis in Mice

**DOI:** 10.3390/cimb48101049

**Published:** 2026-10-10

**Authors:** Gyuri Han, Jaehong Kim, Min Seo Kim, Dong Ho Park, Jong-Sup Bae

**Affiliations:** 1 Research Institute of Pharmaceutical Sciences, Cell & Matrix Research Institute (CMRI), College of Pharmacy, Kyungpook National University, Daegu 41566, Republic of Korea; f11074@naver.com (G.H.); mskim00821@naver.com (M.S.K.); 2 Department of Biochemistry, College of Medicine, Gachon University, Incheon 21999, Republic of Korea; geretics@gachon.ac.kr; 3 Department of Ophthalmology, School of Medicine, Kyungpook National University Hospital, Kyungpook National University, Daegu 41944, Republic of Korea; dongho_park@knu.ac.kr

**Keywords:** picropodophyllotoxin, CLP, sepsis, kidney injury, inflammation

## Abstract

Sepsis is a life-threatening condition caused by a dysregulated host response to infection and is characterized by systemic inflammation, multiorgan dysfunction, and high mortality. Picropodophyllotoxin (PPT), a naturally occurring tyrosine kinase inhibitor derived from the roots of Podophyllum hexandrum, has demonstrated anticancer activity, but its effects on sepsis and the underlying mechanisms remain largely unknown. This study investigated the effects of PPT in a murine model of cecal ligation and puncture (CLP)-induced sepsis. Mice were assigned to six groups: sham control, CLP alone, CLP plus maslinic acid (MA), and CLP plus PPT at 0.5, 1.0, or 2.0 mg/kg. Survival, changes in body weight, inflammatory cytokine levels, and histopathological changes were evaluated. In parallel, human endothelial cells were stimulated with high-mobility group box 1 (HMGB1) and treated with PPT to assess cell viability. Inflammatory markers and protein expression were analyzed using ELISA and Western blotting, respectively. In CLP-induced sepsis, PPT treatment improved survival and attenuated body weight loss compared with untreated controls. PPT also reduced TNF-α, IL-1β, nitric oxide (NO), and other markers of renal inflammation. In HMGB1-stimulated endothelial cells, PPT reduced TLR4 and TNF-α expression and activated PI3K/AKT signaling, with a concomitant increase in cell survival. These findings demonstrate that PPT exerts anti-inflammatory and endothelial-protective effects in experimental sepsis. The observed changes in TLR/NF-κB and PI3K/AKT signaling are correlative, and further studies are required to establish the causal mechanisms involved.

## 1. Introduction

Sepsis is a life-threatening clinical syndrome caused by a dysregulated host response to infection and is characterized by systemic inflammation that can progress to multiorgan dysfunction and failure [1]. Excessive release of proinflammatory mediators, sometimes described as a “cytokine storm,” can contribute to this systemic response. According to the most recent Global Burden of Disease study cited by the authors, an estimated 166 million cases of sepsis and 21.4 million sepsis-related deaths occurred worldwide in 2021. This marked a substantial increase compared with previous years, largely reflecting the effects of the COVID-19 pandemic and reversing earlier declines in sepsis mortality [2]. Sepsis may result from bacterial, fungal, or viral infections and remains associated with high mortality, with reported in-hospital mortality rates of 30–50% [3]. The burden is particularly high among older adults, immunocompromised individuals, and critically ill patients [4], making sepsis a major global health and economic challenge. Pattern recognition receptors (PRRs) are central components of innate immunity that recognize pathogen-associated molecular patterns (PAMPs) and damage-associated molecular patterns (DAMPs) [5]. Excessive activation of these signaling pathways during sepsis can amplify inflammatory responses and contribute to multiple organ failure [5]. Drotrecogin alfa (activated), marketed as Xigris, was previously approved for the treatment of severe sepsis but was withdrawn in 2011 because of limited efficacy and safety concerns [6]. The lack of effective sepsis-specific pharmacological therapies continues to highlight the need for new therapeutic strategies.

Natural products and their bioactive constituents have received increasing attention as potential sources of anti-inflammatory agents [7]. Picropodophyllotoxin (PPT), a structural isomer of podophyllotoxin derived from the roots of *Podophyllum hexandrum*, is a naturally occurring tyrosine kinase inhibitor. PPT has been reported to inhibit insulin-like growth factor 1 receptor signaling in several cancers, including hepatocellular carcinoma, non-small-cell lung cancer, and rhabdomyosarcoma [8,9]. In addition to its anticancer activity, PPT has been associated with antioxidant and anti-inflammatory effects [10,11,12], suggesting that its biological activity may extend beyond cancer-related pathways. 

Cecal ligation and puncture (CLP) is one of the most widely used experimental models of polymicrobial sepsis in rodents and remains an established approach for investigating disease mechanisms and evaluating potential therapeutic interventions [13,14]. Although the model was introduced more than four decades ago, it continues to reproduce key features of polymicrobial sepsis and is widely used in preclinical studies. However, the effects of PPT in sepsis have not been well characterized [13,14]. Therefore, this study evaluated the effects of PPT in a murine model of CLP-induced sepsis and examined whether PPT attenuates sepsis-associated inflammatory responses.

## 2. Materials and Methods

### 2.1. Cell Culture and Reagents

Primary human umbilical vein endothelial cells (HUVECs) were obtained from Cambrex Bio Science (Rockland, ME, USA) and cultured as previously described [15,16]. PPT, 3-(4,5-dimethylthiazol-2-yl)-2,5-diphenyltetrazolium bromide (MTT), dimethyl sulfoxide (DMSO), and maslinic acid (MA) were purchased from Sigma Chemical Co. (St. Louis, MO, USA). MA was used as a positive control because it has been previously characterized as a potent inhibitor of HMGB1-mediated septic responses, demonstrating efficacy in the same HUVEC-based and CLP-induced sepsis models used in the present study [17,18]. The dose of 2 mg/kg was selected based on previously established optimal efficacy in the CLP model, where it significantly improved survival and reduced inflammatory mediator production without observable toxicity [17,18].

### 2.2. Animals and the CLP Procedure

Male C57BL/6 mice aged 8–10 weeks, with a mean body weight of 27 g, were obtained from Orient Bio Co. (Seongnam, Republic of Korea) and housed under standard laboratory conditions (12 h light/dark cycle, 22 ± 2 °C, 50 ± 10% humidity) with free access to food and water. All animal procedures were performed in accordance with the ‘Guide for the Care and Use of Laboratory Animals’ and were approved by the Institutional Animal Care and Use Committee of Kyungpook National University (approval no. KNU 2022-113).

*Anesthesia and analgesia: Mice were anesthetized with isoflurane (3% for induction, 1.5–2% for maintenance) in oxygen via a nose cone. Adequate anesthesia was confirmed by the absence of a pedal withdrawal reflex. Buprenorphine (0.05 mg/kg) was administered subcutaneously before surgery and every 12 h thereafter for 48 h for postoperative analgesia.

*CLP procedure: Sepsis was induced by cecal ligation and puncture (CLP) at moderate or severe intensity according to previously described protocols [19]. Briefly, a 2 cm midline laparotomy was performed to expose and exteriorize the cecum near the ileum. The cecum was ligated 5.0 mm from the tip using 3-0 silk and punctured once with a 22-gauge needle to induce moderate sepsis or twice to induce severe sepsis. A small amount of fecal material was extruded by gently compressing the cecum, which was then returned to the abdominal cavity. The abdominal incision was closed using 4-0 silk. Sham-operated mice underwent the same cecal exteriorization without ligation or puncture.

*Postoperative care and fluid resuscitation: Following surgery, all mice received a subcutaneous injection of pre-warmed sterile saline (1 mL) for fluid resuscitation. Mice were placed on a heating pad (37 °C) until fully recovered from anesthesia and were then returned to their cages with free access to food and water. To minimize suffering, softened chow was provided for the first 48 h after surgery. No antibiotics were administered, consistent with standard CLP protocols in which antibiotic use may confound the assessment of host immune responses.

*Humane endpoints and euthanasia: Mice were monitored twice daily for the first 48 h and once daily thereafter. Humane endpoints were predefined as follows: (i) inability to maintain an upright posture; (ii) loss of >20% of body weight; (iii) severe lethargy or unresponsiveness to gentle stimulation; (iv) labored breathing; or (v) moribund state. Mice meeting any of these criteria were euthanized immediately by CO_2_ inhalation followed by cervical dislocation. For the survival analysis, mice that died spontaneously were recorded as deaths; mice that were euthanized due to humane endpoints were also recorded as deaths.

*Randomization and blinding: Mice were randomly assigned to experimental groups using a computer-generated randomization list. Group allocation was concealed from the investigator performing the CLP surgery by using pre-coded cages. The investigator who administered the test compounds was aware of group allocation (required for dosing), but the investigators who performed the outcome assessments (survival scoring, tissue collection, biochemical assays, and histological analysis) were blinded to group identity. H&E-stained kidney sections were evaluated by two independent, blinded pathologists, and the mean score was used for analysis.

### 2.3. Experimental Design

Two separate animal cohorts were used in this study.

For the survival analysis (Cohort 1), mice were randomly assigned to six experimental groups (n = 20 per group): sham control, CLP alone, CLP + MA (2 mg/kg), and CLP + PPT at 0.5, 1.0, or 2.0 mg/kg. Test compounds or saline vehicle were administered intravenously 24 h after CLP surgery. Survival was monitored for 10 days after CLP and body weight was recorded daily.

For the biochemical and histological analyses (Cohort 2), a separate set of mice was randomly assigned to the same six experimental groups (n = 5 per group). These animals were used exclusively for tissue cytokine measurement, serum marker analysis, and histopathological examination, and were not included in the survival analysis. Test compounds or saline vehicle were administered intravenously 24 h after CLP surgery. Blood samples were collected from the retro-orbital venous plexus at 48 h after CLP (i.e., 24 h after drug administration) and centrifuged at 3000× *g* for 10 min at 4 °C. The resulting serum was aliquoted and stored at −80 °C until analysis. Kidney and liver tissues were collected at 72 h after CLP (i.e., 48 h after drug administration) for cytokine measurement and histopathological examination.

### 2.4. Hematoxylin and Eosin Staining and Histopathological Examination

Twenty-four hours after drug treatment, kidney tissues were fixed in 10% (*v*/*v*) phosphate-buffered formalin for 24 h and embedded in paraffin. Sections 4 μm thick were cut using a microtome, stained with hematoxylin and eosin (H&E), and examined by light microscopy (Leica Microsystems, Wetzlar, Germay) according to standard protocols [15,16]. Semiquantitative histopathological scoring was performed as follows: five randomly selected fields per kidney section (three sections per mouse; n = 5 mice per group) were evaluated at ×200 magnification and scored on a scale of 0 to 4 (0, normal renal architecture with no inflammatory cell infiltration; 1, mild focal infiltration, <10% of field; 2, moderate multifocal infiltration, 10–30% of field; 3, severe diffuse infiltration, >30% of field, with tubular damage; and 4, very severe infiltration with necrosis and loss of renal architecture). H&E-stained kidney sections were evaluated by two independent pathologists who were blinded to group identity, and the mean histopathological score was used for analysis.

### 2.5. Biochemical Measurement

Tumor necrosis factor (TNF)-α and interleukin (IL)-1β levels in liver and kidney tissues were measured using commercial ELISA kits from R&D Systems (Minneapolis, MN, USA). Renal nitric oxide (NO) content was determined using the nitrate reductase method [20,21]. Phosphorylated PI3K levels were measured using an ELISA kit from MyBiosource (San Diego, CA, USA).

### 2.6. Cell Viability Assay

For the cell viability assay, HUVECs were stimulated with HMGB1 (1 μg/mL) for 6 h and then treated with PPT for 48 h, after which cell viability was assessed using the MTT assay [15,16]. For all other HUVEC-based assays (Western blotting and ELISA), cells were stimulated with HMGB1 for 6 h and then treated with PPT for 6 h, as described in the corresponding figure legends.

### 2.7. Western Blot Analysis

Protein extraction and Western blotting were performed as previously described [22], with modifications to specify the protein fraction used for each target. For the analysis of TLR2 and TLR4 (membrane proteins), membrane proteins were extracted using a Mem-PER™ Plus Membrane Protein Extraction Kit (Thermo Fisher Scientific, Waltham, MA, USA) according to the manufacturer’s instructions. For the analysis of NF-κB p65, PI3K p85, p-AKT (Ser473), and total AKT, whole cell lysates were prepared using RIPA buffer supplemented with protease and phosphatase inhibitors. Protein concentrations were determined using the BCA assay (Thermo Fisher Scientific). Equal amounts of protein (20 μg per lane) were separated by 10% SDS-PAGE and transferred to Immobilon-P PVDF membranes (Millipore, Burlington, MA, USA) overnight at 4 °C. The membranes were blocked with 5% non-fat dry milk in Tris-buffered saline containing 0.05% Tween 20 (TBST; 50 mM Tris-HCl, 150 mM NaCl, pH 7.5) for 1 h at room temperature. The membranes were then incubated overnight at 4 °C with primary antibodies against the following proteins: TLR2 (sc-21759, Santa Cruz Biotechnology, Dallas, TX, USA, 1:500), TLR4 (sc-293072, Santa Cruz Biotechnology, 1:500), NF-κB p65 (sc-8008, Santa Cruz Biotechnology, 1:500), PI3K p85α (total, sc-1637, Santa Cruz Biotechnology, 1:500), p-AKT (Ser473) (sc-514032, Santa Cruz Biotechnology, 1:500), total AKT (sc-5298, Santa Cruz Biotechnology, 1:1000), and β-actin (sc-47778, Santa Cruz Biotechnology, 1:2000). After washing with TBST, the membranes were incubated with horseradish peroxidase-conjugated secondary antibodies (anti-mouse or anti-rabbit IgG, 1:5000; Santa Cruz Biotechnology) for 1 h at room temperature. Immunoreactive bands were visualized using enhanced chemiluminescence (ECL) detection reagents (Thermo Fisher Scientific). Band intensities were quantified using the ImageJ Gel Analysis Tool (version 2.x.x/1.5x, NIH, Bethesda, MD, USA), phosphorylated AKT (p-AKT) was normalized to total AKT, and total PI3K p85α was normalized to β-actin as the loading control. Each blot is representative of at least three independent experiments.

### 2.8. Measurement of Tissue Injury Markers

Plasma samples were processed using a commercial Albumin Removal Kit (Thermo Fisher Scientific) to remove albumin. Plasma creatinine and alanine transaminase (ALT) levels were then measured using diagnostic kits from Pointe Scientific.

### 2.9. Statistical Analysis

**Figure 1:**
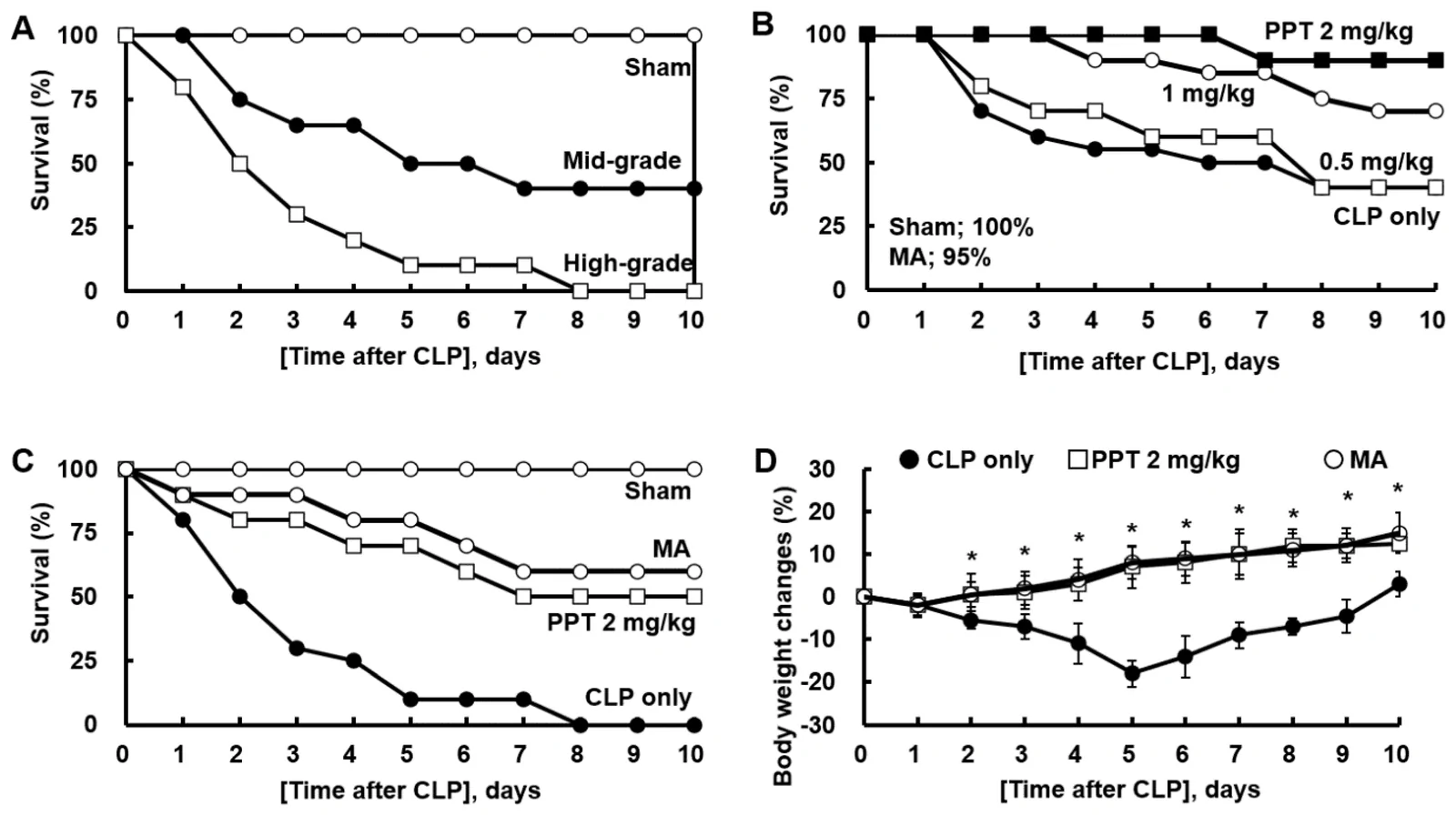
Protective effects of PPT against CLP-induced mortality in mice. (**A**) Survival rates following induction of mid- or high-grade sepsis by CLP. Protective effects of PPT against CLP-induced mortality in mice. (**A**) Survival rates following induction of mid- or high-grade sepsis by CLP. (**B**,**C**) Survival cohort (Cohort 1; n = 20 per group). Each group comprised 20 mice and received an intravenous injection of vehicle (0.5% DMSO), MA (2 mg/kg), or PPT (0.5, 1, or 2 mg/kg) at 24 h after mid-grade (**B**) or high-grade (**C**) CLP. (**B**) Black squares, 2 mg/kg PPT; white circles, 1 mg/kg PPT; white squares, 0.5 mg/kg PPT; black circles, CLP. Survival was monitored for 10 days after CLP. Survival curves were compared using the log-rank (Mantel–Cox) test with Benjamini–Hochberg adjustment for multiple comparisons. Adjusted *p* < 0.01 vs. the CLP group. (**D**) Changes in body weight during the 10-day period shown in (**B**). Data are presented as the mean ± SD (n = 20 per group). Statistical analysis was performed using two-way repeated-measures ANOVA with treatment and time as factors, followed by Tukey’s post hoc test. * Adjusted *p* < 0.05 vs. the CLP group.

All experiments were performed with a minimum of three independent replicates, except for the animal studies where the sample size is specified in the corresponding figure legends. For continuous variables (biochemical and histological data), statistical significance was assessed using one-way analysis of variance (ANOVA), followed by Tukey’s post hoc test for multiple comparisons. A *p* value < 0.05 was considered statistically significant. For survival analysis, Kaplan–Meier survival curves were generated, and differences between groups were compared using the log-rank (Mantel–Cox) test. To account for multiple comparisons across the six experimental groups, the Benjamini–Hochberg false discovery rate (FDR) procedure was applied, and adjusted *p* values are reported. A *p* value < 0.05 was considered statistically significant. For longitudinal body-weight data (Figure 1D), which involved repeated measurements in the same animals over time, a two-way repeated-measures ANOVA was used with treatment (between-subjects factor) and time (within-subjects factor) as the two factors, including their interaction term. When Mauchly’s test indicated a violation of the sphericity assumption, the Greenhouse–Geisser correction was applied. Post hoc comparisons were performed using Tukey’s test for multiple comparisons. A *p* value < 0.05 was considered statistically significant. All statistical analyses were performed using SPSS software (version 16.0; SPSS Inc., Chicago, IL, USA) or GraphPad Prism (version 8.0; GraphPad Software, San Diego, CA, USA).

## 3. Results

### 3.1. Protective Effects of PPT on CLP-Induced Sepsis

The mortality associated with CLP-induced sepsis varies with the severity of the procedure, which is influenced by the site and extent of cecal puncture [19]. Survival curves for mice subjected to mid- or high-grade CLP are shown in Figure 1A. Consistent with previous findings [19], high-grade CLP resulted in 100% mortality, whereas 40% of mice survived after mid-grade CLP. Both models were used to evaluate the effects of PPT. Mice received PPT at 0.5, 1.0, or 2.0 mg/kg, with 0.5% DMSO and MA (2 mg/kg) serving as negative and positive controls, respectively [17,18]. In the mid-grade CLP model, PPT at 1.0 and 2.0 mg/kg increased 10-day survival to 70% and 90%, respectively (Figure 1B). PPT at 0.5 mg/kg did not significantly improve survival compared with the control, whereas survival reached 95% in the MA group (Figure 1B). Based on these findings, 2.0 mg/kg PPT was selected for subsequent analyses. In the high-grade CLP model, in which untreated mice exhibited 100% mortality (Figure 1A), 2.0 mg/kg PPT significantly improved survival compared with untreated animals (log-rank test with Benjamini–Hochberg adjustment, adjusted *p* < 0.01; Figure 1C). Mid-grade CLP also reduced body weight by up to 18% from baseline, whereas treatment with 2.0 mg/kg PPT resulted in a recovery of up to 25% by day 5 after surgery compared with the vehicle-treated CLP group.

### 3.2. Suppressive Effects of PPT on Inflammatory Cytokine Levels and Renal Inflammation in CLP-Operated Mice

**Figure 2:**
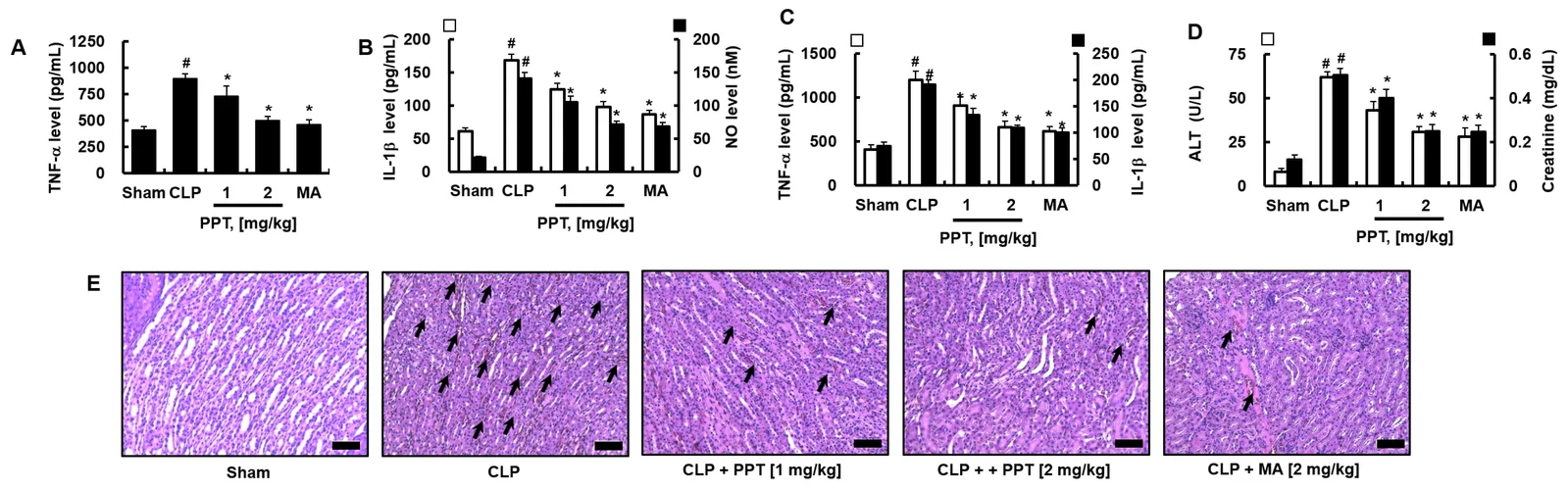
Effects of PPT on inflammatory mediator levels and renal and hepatic injury following CLP. Biochemical/histological cohort (Cohort 2; n = 5 per group). Each group comprised five mice and received an intravenous injection of vehicle (0.5% DMSO), MA (2 mg/kg), or PPT (1 or 2 mg/kg) at 24 h after high-grade CLP. Kidney and liver tissues were collected at 72 h after CLP (i.e., 48 h after drug administration) and homogenized for measurement of TNF-α (**A**,**C**) and IL-1β (**B**,**C**). (**D**) Plasma ALT and creatinine levels were measured at 72 h after CLP. (**E**) Kidney tissues were stained with H&E at 72 h after CLP. Arrows indicate leukocyte infiltration. Representative microscopic images of kidney tissue at 72 h after CLP are shown (original magnification, 200×; scale bar, 160 μm). Semiquantitative histopathological scoring of inflammatory cell infiltration (0–4 scale) was performed by two independent, blinded pathologists. Data are presented as the mean ± SD (n = 5 per group). Statistical significance was assessed using one-way ANOVA followed by Tukey’s post hoc test. # Adjusted *p* < 0.01 vs. the sham control group; * adjusted *p* < 0.01 vs. the CLP group.

We next evaluated the effects of PPT on inflammatory mediators and organ injury following CLP. In the high-grade CLP model, at 72 h after CLP (i.e., 48 h after drug administration), PPT treatment significantly reduced renal levels of TNF-α, IL-1β, and nitric oxide (NO), consistent with attenuation of renal inflammation [23] (Figure 2A,B). PPT also reduced TNF-α and IL-1β concentrations in the liver (Figure 2C). Because systemic inflammation during sepsis can cause organ injury and contribute to multiple organ failure [1], we further examined markers of hepatic and renal injury. Serum ALT and creatinine levels were markedly increased after CLP surgery, and PPT treatment significantly attenuated these increases (Figure 2D). Histological examination of kidney tissue by H&E staining further showed reduced inflammatory cell infiltration following PPT treatment compared with the untreated group (Figure 2E). Semiquantitative scoring of inflammatory cell infiltration (0–4 scale) confirmed that CLP significantly increased the histopathological score compared with sham controls (3.2 ± 0.4 vs. 0.3 ± 0.2, respectively; *p* < 0.01), whereas PPT treatment at 1 and 2 mg/kg significantly reduced the score to 2.1 ± 0.3 and 1.4 ± 0.3, respectively (*p* < 0.01 vs. CLP). Together, these findings indicate that PPT attenuates CLP-induced inflammatory responses and associated organ injury.

### 3.3. Inhibitory Effects of PPT on TLR and NF-κB Expression

**Figure 3:**
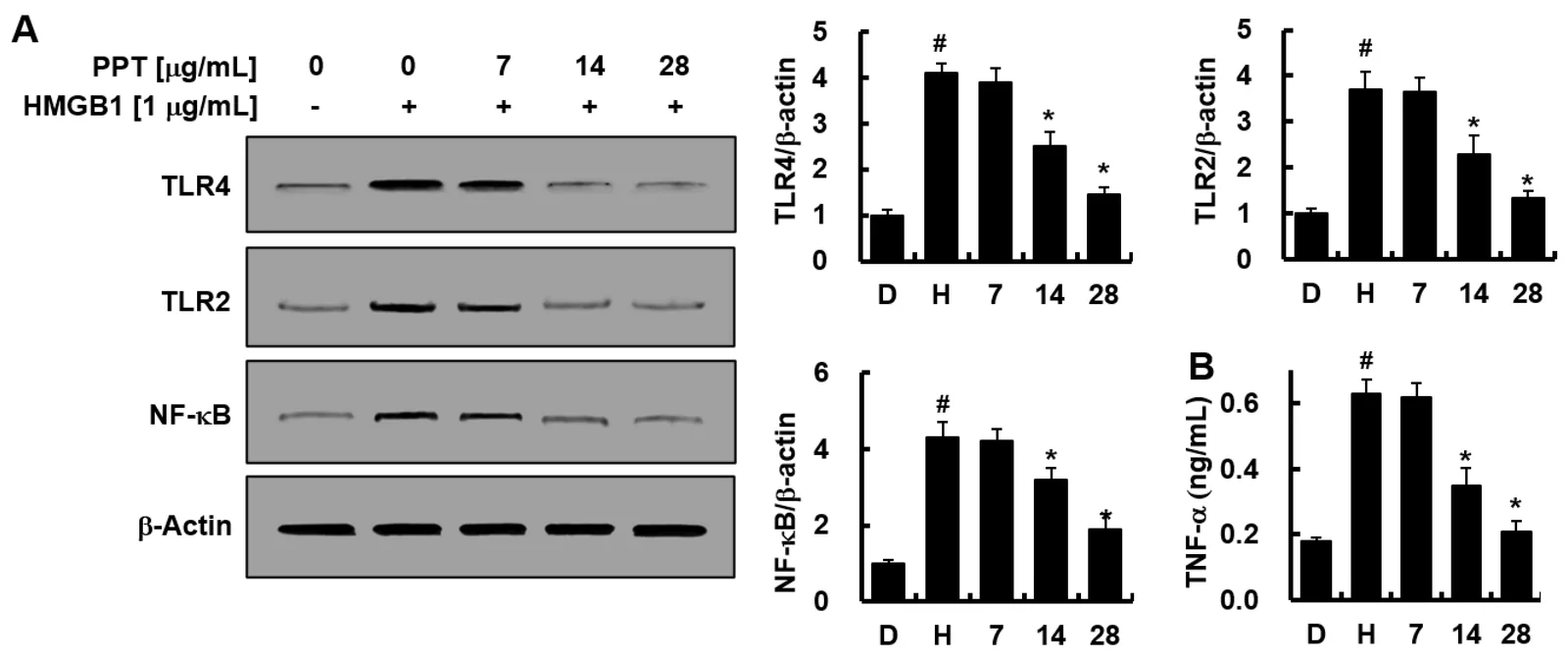
Effects of PPT on HMGB1-induced TLR/NF-κB signaling. (**A**) HUVECs were treated with HMGB1 (1 μg/mL) for 6 h and then treated with various concentrations of PPT for an additional 6 h. Cell lysates were subjected to Western blotting to determine TLR4, TLR2, and NF-κB (p65) protein levels. β-Actin was used as the loading control. Representative Western blot images (**left**) and densitometric quantification of relative band intensities normalized to β-actin (**right**) are shown. Molecular weight markers are indicated on the left of each blot. (**B**) Supernatants from the cells described in (**A**) were analyzed for TNF-α by ELISA. Values are presented as the mean ± SD of three independent experiments. D, 0.5% DMSO vehicle control; H, HMGB1. ^#^ *p* < 0.01 vs. DMSO; * *p* < 0.01 vs. the HMGB1 group.

Primary HUVECs were stimulated with HMGB1, a mediator involved in the late phase of sepsis [24], and then treated with PPT for 6 h. TLR expression was assessed by Western blotting. PPT at 14 or 28 µg/mL significantly reduced TLR2 and TLR4 expression compared with HMGB1-stimulated cells (Figure 3A). At the same concentrations, PPT also reduced NF-κB expression, a downstream component of TLR signaling (Figure 3A). The in vivo doses of 1 and 2 mg/kg were selected empirically, based on the effective concentration range identified in vitro (14–28 μg/mL), and were validated by the survival and tissue analyses presented in Figure 1 and Figure 2. For approximate contextual comparison only, these in vivo doses were converted to nominal plasma concentrations of approximately 14 and 28 μg/mL, respectively, assuming an estimated mouse blood volume of 72 mL/kg [25] and an average body weight of 27 g (total blood volume ≈ 2 mL). This calculation represents a simple dose-to-volume approximation and does not account for pharmacokinetic parameters such as absorption, distribution, plasma protein binding, metabolism, or clearance. Therefore, it should not be interpreted as evidence of pharmacokinetic equivalence between the in vitro and in vivo settings. PPT treatment also significantly reduced TNF-α production in HUVECs (Figure 3B), consistent with suppression of HMGB1-induced inflammatory signaling. Together, these results show that PPT reduces TLR2, TLR4, and NF-κB expression and TNF-α production in HMGB1-stimulated endothelial cells.

### 3.4. Effects of PPT on Cell Survival In Vitro

**Figure 4:**
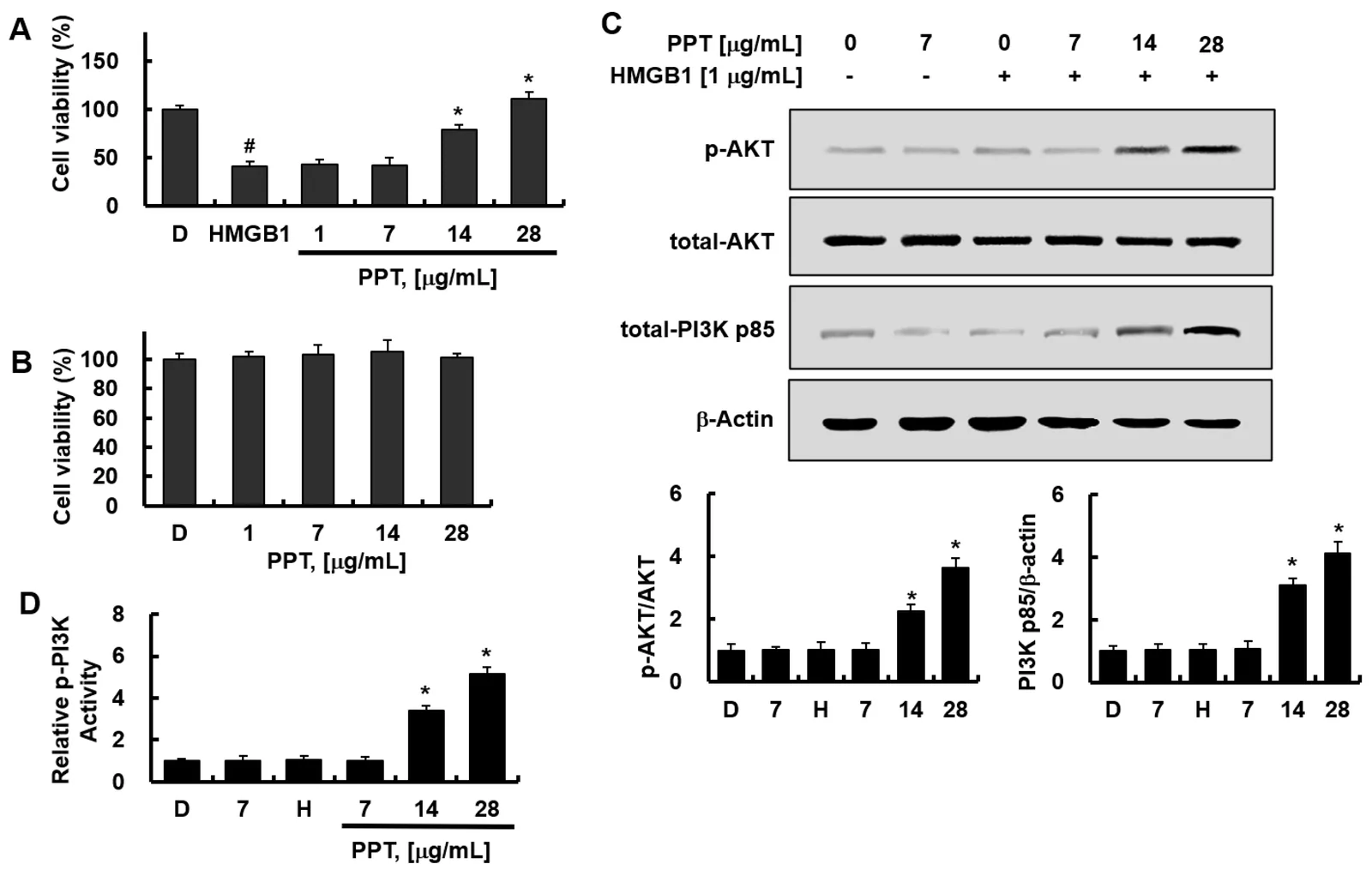
Effects of PPT on cell viability and PI3K/AKT signaling during HMGB1-induced inflammatory stimulation. HUVECs were incubated with (**A**) or without (**B**) HMGB1 (1 μg/mL) for 6 h and then treated with various concentrations of PPT for an additional 6 h for Western blotting and ELISA (**C**,**D**), or for 48 h for the MTT cell viability assay (**A**,**B**). (**A**,**B**) Cell viability was assessed using the MTT assay. (**C**) Whole cell lysates were subjected to Western blotting to determine and total PI3K p85α, p-AKT (Ser473), and total AKT protein levels. p-AKT was normalized to total AKT, and total PI3K p85α was normalized to β-actin as the loading control. Representative Western blot images (**left**) and densitometric quantification of relative band intensities normalized to β-actin (**right**) are shown. Molecular weight markers are indicated on the left of each blot. (**D**) Phosphorylated PI3K levels were measured by ELISA. Values are presented as the mean ± SD of three independent experiments. D, 0.5% DMSO vehicle control; H, HMGB1. ^#^ *p* < 0.01 vs. DMSO; * *p* < 0.01 vs. the HMGB1 group.

Previous studies have reported that PPT can regulate inflammatory responses and induce apoptosis in cancer cells through pathways involving ROS generation, p38 MAPK activation, and STAT3 inhibition [10,11,12]. We therefore examined the effects of PPT on HUVEC viability following HMGB1 stimulation. HMGB1 reduced HUVEC viability, whereas treatment with PPT at 14 or 28 µg/mL significantly increased cell viability (Figure 4A). PPT alone did not significantly affect HUVEC viability (Figure 4B). Total PI3K p85α levels were unchanged following HMGB1 stimulation but increased after PPT treatment (Figure 4C). PPT treatment was also associated with an increased p-AKT/total AKT ratio (Figure 4C) and increased phosphorylated PI3K (p-PI3K) levels as measured by ELISA (Figure 4D), whereas total AKT levels remained unchanged (Figure 4C). The increase in the p-AKT/total AKT ratio indicates enhanced AKT activation rather than a change in total AKT abundance. These findings suggest that the PPT-associated increase in HUVEC viability is accompanied by activation of PI3K/AKT signaling.

## 4. Discussion

Sepsis is a highly heterogeneous syndrome caused by a dysregulated host response to infection and can progress to multiorgan failure [1]. It can result from bacterial, viral, or fungal infections and is associated with substantial mortality among critically ill patients in intensive care units [26]. Proinflammatory mediators, including TNF-α, IL-1β, and IL-6, play important roles in the pathogenesis of sepsis [26,27]. Drotrecogin alfa (activated), marketed as Xigris, was previously approved for severe sepsis but was withdrawn from the global market in 2011 after a large trial failed to demonstrate a survival benefit in patients with septic shock [6]. The continued lack of effective sepsis-specific pharmacological therapies underscores the need to identify new therapeutic strategies. In the present study, PPT reduced NO production and the levels of inflammatory cytokines, including TNF-α and IL-1β, in mice with CLP-induced sepsis. 

Following CLP, PPT treatment reduced TNF-α and IL-1β levels, suppressed NO production, and attenuated renal inflammation, supporting an anti-inflammatory effect in vivo. In HMGB1-stimulated human endothelial cells, PPT reduced TLR4 expression and TNF-α production and was associated with activation of PI3K/AKT signaling and increased cell viability. Together, these findings suggest that PPT may influence both inflammatory signaling and endothelial cell survival during septic challenge. The improvement in survival observed in PPT-treated mice further supports its potential protective effects in experimental sepsis. However, because these findings were obtained in a murine CLP model and an HMGB1-stimulated endothelial cell system, the therapeutic relevance of PPT to human sepsis requires further investigation. 

To investigate the molecular mechanisms associated with the effects of PPT on inflammatory responses, we assessed TLR2, TLR4, and NF-κB expression in HUVECs. Toll-like receptors regulate inflammatory signaling and cytokine production during sepsis, and increased TLR2 and TLR4 expression has been associated with adverse outcomes [28]. NF-κB is an important downstream mediator of TLR signaling and contributes to inflammatory responses during sepsis [29]. In the present study, HMGB1 stimulation increased TLR2, TLR4, and NF-κB expression, whereas PPT treatment attenuated these increases. In parallel, PPT reduced renal inflammation and improved survival in the CLP model. These findings suggest that suppression of TLR/NF-κB signaling may contribute to the anti-inflammatory effects of PPT observed in this experimental setting.

The PI3K/AKT signaling pathway is an important regulator of cell survival [30]. In HMGB1-stimulated HUVECs, PPT treatment was associated with an increased p-AKT/total AKT ratio and increased total PI3K p85α levels, concomitant with improved cell viability. In addition, phosphorylated PI3K (p-PI3K) levels measured by ELISA were increased following PPT treatment (Figure 4D). The increase in the p-AKT/total AKT ratio indicates enhanced AKT activation rather than a change in total AKT abundance. However, this observation appears to contrast with PPT’s established pharmacology as an IGF-1R inhibitor, which would be expected to suppress rather than activate PI3K/AKT signaling [8,9,10]. Several possible explanations may account for this apparent discrepancy, including: (i) cell-type-dependent differences in IGF-1R dependency between cancer cells and primary endothelial cells; (ii) a compensatory pro-survival response to HMGB1-induced stress that is independent of IGF-1R; (iii) off-target or pleiotropic effects of PPT at the concentrations used; (iv) indirect modulation of PI3K/AKT through HO-1 or redox-dependent pathways; or (v) temporal dissociation between early IGF-1R inhibition and later compensatory AKT activation. Importantly, none of these hypotheses have been experimentally validated in the present study, and IGF-1R dependency was not directly assessed.

The increase in the p-AKT/total AKT ratio indicates enhanced AKT activation rather than a change in total AKT abundance. However, the present data demonstrate only a correlative relationship and do not establish a causal role for PI3K/AKT signaling in mediating the protective effects of PPT. The observed increase in PI3K/AKT signaling may represent a downstream consequence of PPT treatment, a compensatory response to HMGB1-induced stress, or an epiphenomenon unrelated to the primary mechanism of action. Because PI3K/AKT signaling also participates in the regulation of inflammatory responses, its activation could potentially contribute to the effects of PPT on cell survival and inflammation, but this hypothesis requires direct experimental validation. Future studies employing PI3K/AKT-specific inhibitors (e.g., LY294002, wortmannin) or genetic knockdown (e.g., AKT siRNA) will be necessary to determine whether PI3K/AKT activation is causally required for the protective effects of PPT.

This study focused primarily on the effects of PPT on inflammatory mediator production, endothelial cell viability, and PI3K/AKT signaling. However, sepsis is a complex syndrome involving multiple signaling pathways, immune cell populations, and organ-specific responses, and additional mechanisms may contribute to the effects observed here. Several limitations should also be considered. First, this study relied on a CLP-induced murine model, and findings from this model may not fully reflect the heterogeneity of human sepsis. Second, more comprehensive dose–response and toxicological studies are needed to characterize the efficacy and safety of PPT. Third, signaling pathways other than PI3K/AKT were not examined in detail, and interspecies differences may further limit translation of these findings. Additional mechanistic and preclinical studies are therefore warranted before the potential efficacy and safety of PPT in human sepsis can be evaluated.

The present study has several limitations. First, although we observed an association between PPT treatment, increased PI3K/AKT signaling, and improved endothelial cell viability, these data do not establish a causal role for PI3K/AKT in mediating the protective effects of PPT. Pathway-specific inhibitors or genetic knockdown experiments would be required to determine whether PI3K/AKT activation is necessary for the observed effects. Second, the relationship between the in vivo doses (mg/kg) and in vitro concentrations (μg/mL) used in this study is only approximate. The conversion based on estimated blood volume does not account for pharmacokinetic processes such as absorption, distribution, plasma protein binding, metabolism, and clearance. Therefore, the in vitro and in vivo exposures should not be regarded as pharmacokinetically equivalent, and the in vivo doses should be considered independently optimized and empirically validated in the CLP model.

## 5. Conclusions

Overall, PPT treatment improved survival, reduced inflammatory mediator production, attenuated renal inflammation, and enhanced endothelial cell viability in the experimental models used in this study. These effects were accompanied by, but not necessarily mediated by, reduced TLR/NF-κB signaling and increased PI3K/AKT signaling. The present study establishes an association between PPT treatment and modulation of these pathways, but does not demonstrate a causal relationship. In particular, the observation of increased PI3K/AKT signaling appears to contrast with PPT’s established IGF-1R-inhibitory pharmacology, and this discrepancy remains unresolved. Taken together, the findings support further investigation of PPT as a potential modulator of inflammatory responses and endothelial cell survival in sepsis, with future studies needed to define (i) the precise mechanistic contribution of PI3K/AKT signaling and (ii) the relationship between PI3K/AKT activation and IGF-1R inhibition.

## Data Availability

The data presented in this study are available upon reasonable request from the corresponding author.

## Abbreviations

The following abbreviations are used in this manuscript:

PPT	picropodophyllotoxin
CLP	cecal ligation and puncture
HMGB1	high-mobility group box 1
NO	nitric oxide
HUVECs	human umbilical vein endothelial cells
IL	interleukin
MTT	3-(4,5-dimethylthiazol-2-yl)-2,5-diphenyltetrazolium bromide
DMSO	dimethyl sulfoxide
